# The Experiences of Women With Polycystic Ovary Syndrome of the Healthcare They Receive: A Qualitative Systematic Review

**DOI:** 10.1111/jan.70265

**Published:** 2025-09-30

**Authors:** Baoying Zhang, Joan Lalor

**Affiliations:** ^1^ School of Nursing & Midwifery Trinity College Dublin Dublin Ireland

**Keywords:** caregiving, delivery of health care, literature review, obstetrics and gynaecology, public health nursing, qualitative approaches, women's health

## Abstract

**Background:**

More than 12% of women worldwide are affected by polycystic ovary syndrome (PCOS), whose symptoms are similar to those of puberty, often leading to delayed diagnosis and missing the opportunity for early intervention. This not only puts PCOS women under physical and mental stress but also reduces their trust in doctors and makes them dissatisfied with the healthcare they receive, which in turn affects their quality of life. Therefore, to improve the doctor‐patient relationship and promote health, it is essential to investigate and understand the healthcare experiences that women with polycystic ovary syndrome (PCOS) receive.

**Aim:**

To explore the experiences of women with PCOS when they receive healthcare.

**Design:**

Qualitative systematic review.

**Methods:**

Data were collected and screened using the systematic review management system Covidence, based on the established inclusion criteria. The Critical Appraisal Skills Programme Checklist was used for critical appraisal, and thematic analysis was used for data analysis.

**Data Sources:**

The databases searched included CINAHL, MEDLINE, PsycINFO, and Scopus. The search was limited to studies published in English between 2002 and May 2024.

**Results:**

Seven studies were selected for final inclusion. Three themes were identified: (1) responsive care from healthcare practitioners, (2) managing polycystic ovarian syndrome, and (3) polycystic ovary syndrome and its impact on self‐image.

**Conclusion:**

The development of a multidisciplinary PCOS clinic, the establishment of online support groups, and the creation of comprehensive patient‐centered treatment plans are vital to enhancing the health outcomes of women with PCOS.

**Impact:**

Multidisciplinary PCOS clinics, online support groups, and comprehensive patient‐centered treatment plans can improve health outcomes for women with PCOS.

**Reporting Method:**

The EQUATOR guidelines for PRISMA have been utilised.

**Patient or Public Contribution:**

No patient or public contribution.

## 1 Introduction

Polycystic ovary syndrome (PCOS) is a common endocrine condition that affects more than 12% of women worldwide during their reproductive years (World Health Organization 2023). The symptoms of PCOS are wide‐ranging and varied, including irregular menstruation, hirsutism, infertility, obesity, hair loss, metabolic problems, cardiovascular disease, etc. (Ahmadi et al. 2020; Dybciak et al. 2022; Yang et al. 2021). These symptoms and complications not only affect the quality of life and overall health of women with PCOS from adolescence to menopause but also have a significant impact on their psychological, metabolic, and reproductive health, as well as their overall well‐being (Ahmadi et al. 2020). Women with PCOS often report issues such as obesity, acne, poor self‐image, and excessive hair growth, which significantly impact their lives, leading to decreased self‐esteem and self‐acceptance, and an increased risk of anxiety and depression (Wang et al. 2023). In addition, many women with PCOS experience mental health problems following their diagnosis, particularly when informed of their potential risk of infertility or after unsuccessful attempts to conceive (Dybciak et al. 2022; Sukhapure et al. 2022). These problems include depression, anxiety, schizophrenia, bipolar disorder, and other psychological conditions (Brutocao et al. 2018; Douglas et al. 2022; Tan et al. 2017). Several studies have indicated that women with PCOS are at a higher risk for mental health issues such as anxiety and depression (Dybciak et al. 2022; Greenwood et al. 2018; Kocak and Ugurlu 2022). This finding was further supported by a controlled study conducted by Ahmadi et al. (2020), which found that infertile women with PCOS were at a higher risk of six personality disorders (sadistic, schizotypal, depressive, avoidant, negative, and antisocial) and three severe personality disorders (schizotypal, borderline, and paranoid) compared to infertile women without PCOS. However, the mental health issues of women with PCOS are often ignored by healthcare professionals (Ismayilova and Yaya 2023; Sourouni et al. 2024). This neglect is largely due to the lack of information provided by healthcare practitioners (Crete and Adamshick 2011; Ismayilova and Yaya 2023, 2022a, 2022b; Soucie et al. 2021; Sydora et al. 2023; Tomlinson et al. 2017), resulting in many women lacking information about PCOS management and treatment and its potential risks. In the absence of professional guidance, they often feel ignored and misunderstood, and gradually lose trust in the healthcare system, which not only increases the psychological burden but also further exacerbates disappointment and dissatisfaction with the healthcare system.

Research shows that women with PCOS are generally dissatisfied with the healthcare services they receive (Gibson‐Helm et al. 2017; Hoyos et al. 2020; Ismayilova and Yaya 2022a). Specifically, these women with PCOS report that the information and support provided by their physicians were often inadequate and did not meet their urgent needs for effective management of PCOS symptoms (Hoeger et al. 2021). Furthermore, Wang et al. (2023) further revealed that more than 50% of the participants expressed a strong desire for professional consultation and guidance on lifestyle and diet management, but these services were not provided to them. Despite growing research on PCOS, the mental health status of women with PCOS and their perceptions of healthcare services remain insufficiently understood (Ahmadi et al. 2020). Therefore, it is necessary to further investigate the gaps in service delivery among healthcare providers better to address the health issues of women with PCOS and improve their mental health.

Lifestyle changes and oral contraceptives are widely recognised as effective methods for managing and controlling the symptoms of PCOS (National Institute of Child Health and Human Development, n.d.). However, despite the proven effectiveness of these approaches, health management for women with PCOS remains a significant challenge. Specifically, support and advice on dietary and lifestyle changes for women with PCOS often lack sustainability and often fail to meet the actual needs of this specific population (Hoeger et al. 2021). To date, research on the healthcare experiences of women with PCOS remains relatively limited (Elghobashy et al. 2023). Furthermore, the health and healthcare needs of women with PCOS in different regions and healthcare settings have not been systematically studied (Sydora et al. 2023). This lack of insight into the healthcare experiences and attitudes of women with PCOS prevents healthcare providers from fully understanding the challenges these women face, the significance of healthcare to them, and the types of services they expect. As a result, healthcare services often fail to meet their needs effectively, leading to inefficient healthcare delivery and wasted resources. Moreover, this disconnect may exacerbate women's distrust of healthcare providers, further compromising their overall health and well‐being. Consequently, it has become imperative to further explore the healthcare experiences of women with PCOS. This will not only help us gain a more complete understanding of the healthcare experiences of women with PCOS but also assist in developing responsive, patient‐centered care plans for them, thereby improving their overall health and enhancing their trust and satisfaction with healthcare.

## 2 Aims

The systematic review aimed to explore the experiences of women with PCOS when they receive healthcare.

## 3 Methods

### 3.1 Design

This qualitative systematic review was conducted following the ENTREQ statement (Tong et al. 2012), which aims to provide a framework for reviewers and researchers to report their systematic review work more comprehensively and clearly. The PEO (population, exposure, and outcome) mnemonic was used to construct the research questions for this qualitative systematic review. It was stated as follows: What are the experiences (O) of women with polycystic ovary syndrome (P) of the healthcare they receive (E)? This review was not registered in PROSPERO.

### 3.2 Search Strategy

Four databases, including CINAHL, MEDLINE, PsycINFO, and Scopus, were systematically searched on February 18, 2024, and updated on May 1, 2024. These databases were chosen as they were considered closely related to the life sciences and biomedical fields. Literature was limited to publications after 2002, as the earliest documented article investigating the experiences of women with PCOS was published in 2002 (Kitzinger and Willmott 2002). In addition, backward citation searches of the included studies were conducted to identify additional studies. Keywords included ‘polycystic ovary syndrome’, ‘healthcare’, and ‘experience or perception’. Search terms or keywords were combined using the Boolean operators ‘OR’ and ‘AND’, and the following terms were searched: ‘Stein‐Leventhal Syndrome’ OR ‘Sclerocystic Ovary Syndrome’ OR ‘Polycystic Ovarian Syndrome’ OR PCOS OR ‘Polycystic Ovary Syndrome’ OR Ovaries OR ‘Polycystic Ovary Disease’ OR ‘Stein Leventhal Syndrome’ OR ‘Polycystic Ovarian Disease’ OR ‘Sclerocystic Ovarian Degeneration’ OR ‘Sclerocystic Ovaries’ OR ‘Sclerocystic Ovary’ AND ‘Healthcare Utilisation’ OR ‘Healthcare Delivery’ OR ‘Primary care’ OR ‘Primary Healthcare’ OR ‘Delivery of Healthcare’ OR Treatment OR ‘Health Care Delivery’ OR ‘Healthcare Deliveries’ OR Support OR ‘Deliveries, Healthcare’ OR ‘Delivery, Healthcare’ OR ‘Healthcare Resource Utilisation’ OR ‘Delivery, Health Care’ OR ‘Health Care’ OR Healthcare OR ‘Personal Health Services’ OR ‘Healthcare Services Accessibility’ OR ‘Healthcare Services’ OR ‘Healthcare Access’ OR ‘Medical Consultations’ AND Experience OR Qualitative OR ‘User Experience’ OR ‘Sensory Processing’ OR ‘Processing, Sensory’ OR Feelings OR Perceptions OR ‘Lived Experiences’ OR Perspectives OR Opinions OR ‘Lived Experience’ OR Phenomenology OR ‘Qualitative Research’ OR ‘Follow‐up Care’ OR ‘Support Experience’ OR Beliefs OR Attitudes OR Views OR ‘Experiences of Patients’.

### 3.3 Inclusion and Exclusion Criteria

In this systematic review, original studies using qualitative, survey, and qualitative data extracted from mixed‐method studies to explore the views, attitudes, and experiences of women with PCOS in the healthcare they receive were included. The search was limited to studies published in English and restricted to studies published between 2002 and May 2024. Table 1 outlines the inclusion and exclusion criteria.

**TABLE 1:** The inclusion and exclusion criteria.

	Inclusion	Exclusion
Population	Women diagnosed with PCOS through formal clinical examination; > 18 years old	Women self‐reported the presence of PCOS symptoms but lack of formal diagnosis by medical staff; < 18 years old
Exposure	The experiences of care and support from healthcare	Experience of care and support not for PCOS
Outcome	All perceptions, attitudes, views, experiences, feelings, feedback, remarks, and suggestions made by women with PCOS toward the healthcare they experienced and received	Studies that made no mention of attitudes, experiences, beliefs, views, perceptions, opinions, outlooks, and feelings
Type of study	Qualitative reviews; Primary studies; Surveys; Qualitative data in mixed method studies were also extracted	Quantitative studies; Letters; Opinion; Literature reviews; Commentaries; Years prior to 2002

### 3.4 Search Outcomes

**FIGURE 1:**
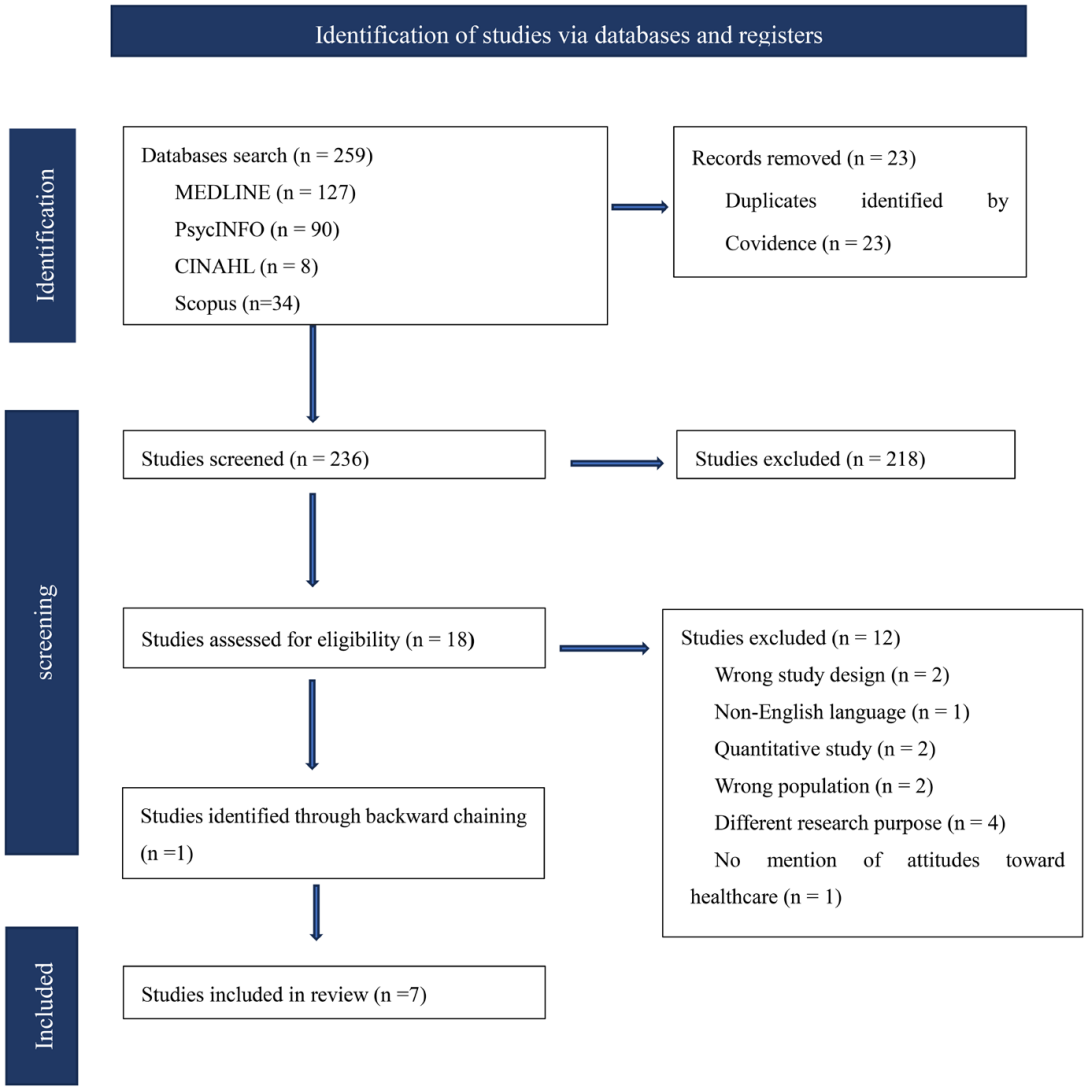
PRISMA flowchart.

The initial search for studies was conducted independently by the first author (BYZ). All identified citations were managed using the systematic review management system Covidence. A total of 259 studies were identified from the database search, and after removing 23 duplicates, 236 studies were retained for further screening. During the title and abstract screening stage, 218 studies were excluded based on the established inclusion criteria, and 18 full‐text papers were identified for further screening. During the full‐text review stage, 12 studies were deemed ineligible and excluded, and the remaining 6 studies were deemed eligible for review. In addition, 1 study was identified in the backward‐linked included studies. Finally, 7 studies were included in this review. PRISMA flowchart outlines the study results (Figure 1).

### 3.5 Quality Appraisal

The Critical Appraisal Skills Programme Checklist (CASP 2018) was used to appraise the overall methodological quality of this review. The CASP checklist was chosen since it was considered suitable for assessing articles for qualitative research (Tong et al. 2012), which provides a uniform structure for appraising the methodological rigour of diverse research studies, guaranteeing consistent evaluation among different studies and reviewers (Long et al. 2020). As Moorley and Cathala (2019) point out, when evaluating qualitative research, attention should be paid to the transparency, credibility, and authenticity of the research to ensure its rigour. The CASP checklist consists of 10 items covering aspects of study design, sampling, data collection, reflexivity, ethical issues, data analysis, results, and research value. The assessments are rated on a scale of 0 (lowest quality) to 10 (highest quality). The evaluation was completed by the primary reviewer (BYZ) and confirmed by the secondary reviewer (JL). During the evaluation process, any disagreements were resolved through discussion. Finally, 4 studies were classified as high‐quality studies, while 3 studies were rated as moderate‐quality studies. Table 2 shows the evaluation results of CASP in detail.

**TABLE 2:** Critical Appraisal Skills Programme (CASP) result.

Citation	Q1	Q2	Q3	Q4	Q5	Q6	Q7	Q8	Q9	Q10	Score
Ismayilova and Yaya (2022a)	Y	Y	Y	Y	Y	Y	Y	Y	Y	Y	10
Ismayilova and Yaya (2022b)	Y	Y	Y	Y	Y	Y	N	Y	Y	Y	9
Ismayilova and Yaya (2023)	Y	Y	Y	Y	Y	Y	N	Y	Y	Y	9
Tomlinson et al. (2017)	Y	Y	C	Y	Y	C	Y	Y	Y	Y	8
Soucie et al. (2021)	Y	Y	Y	N	Y	N	Y	C	Y	Y	7
Crete and Adamshick (2011)	Y	Y	Y	Y	Y	C	Y	C	C	Y	7
Sydora et al. (2023)	Y	Y	Y	C	Y	C	Y	C	Y	Y	7

*Note:* The CASP Checklist for Qualitative Research: Q1 = Was there a clear statement of the aims of the research? Q2 = Is a qualitative methodology appropriate? Q3 = Was the research design appropriate to address the aims of the research? Q4 = Was the recruitment strategy appropriate to the aims of the research? Q5 = Was the data collected in a way that addressed the research issue? Q6 = Has the relationship between the researcher and participants been adequately considered? Q7 = Have ethical issues been taken into consideration? Q8 = Was the data analysis sufficiently rigorous? Q9 = Is there a clear statement of findings? Q10 = How valuable is the research?

Abbreviations: C: can't tell; N, no; Y, yes.

### 3.6 Data Extraction

One reviewer (BYZ) performed data extraction for the included studies using a predefined data extraction form, including study aim, study design, study setting/location, sampling method, exclusion and inclusion criteria, number of participants, recruited sample, data collection and analysis methods, and findings. Any discrepancies were resolved through discussion at team meetings.

### 3.7 Data Synthesis

The thematic analysis framework was used for data analysis, and its strengths lie in its flexibility and simplicity. It provides a direct and rigorous approach, helping researchers to conduct precise step‐by‐step data analysis and present a rich and detailed perspective on the data (Braun and Clarke 2019, 2006). The data went through six processes, including: (1) familiarity with data; (2) generating initial codes; (3) searching for themes; (4) reviewing themes; (5) defining and naming themes; and (6) producing the report. In recent years, Braun and Clarke (2019) further proposed ‘reflective thematic analysis’, emphasising the subjectivity and reflexivity of researchers in the analysis process and indicating that variations of thematic analysis methods are acceptable if rationalised.

## 4 Results

### 4.1 Characteristics of Included Studies

A total of 7 studies were included in this review, of which 71.43% were published in the past 5 years. The studies were mainly from Canada (*n* = 5), and the rest were from the United States (*n* = 1) and the United Kingdom (*n* = 1). Sample sizes varied depending on the study design, ranging from a small‐scale interview with 10 people to a large‐scale cross‐sectional survey of 296 people. It is worth noting that 6 studies (85.71%) reported the ethnic background of the participants, and the results showed that the sample structure was obviously unbalanced, with the proportion of White/Caucasian participants as high as 70% to 84.5%, see Table 3.

**TABLE 3:** Study characteristics.

Author/Year/Study location	Purpose of study	Participants	Ethnicity (%)	Data collection method	Data analysis method
Tomlinson et al. (2017) The United Kingdom	To explore the impact of the diagnosis of polycystic ovary syndrome (PCOS) on health/ill health identity, the diagnostic experience and health belief of women with PCOS	32 premenopausal adult women with PCOS (diagnosis confirmed by Rotterdam criteria); Ages 18–45 years	N/A	Questions and semi‐structured group discussion	Thematic analysis
Ismayilova and Yaya (2022a) Canada	To explore the perceptions and experiences of PCOS diagnosis in Canada	296 participants attended the online survey; 25 women attended the following interview; Age ≥ 18 years	White/Caucasian (80.1), Asian/Pacific Islander (8.2), Black (2.4), Indigenous (2.1), Hispanic/Latino (0.7) and other (6.5)	Online questionnaire and semi‐structured interview	Thematic analysis
Ismayilova and Yaya (2022b) Canada	To explore women's experiences navigating the healthcare system and their insights on what would be improved based on their lived experience	Age ≥ 18 years; 25 participants who reported a medical diagnosis of PCOS	Black (4), East Asian (8), Middle Eastern (8), South Asian (8), and White/Caucasian (72)	In‐depth and semi‐structured phone interviews	Thematic analysis
Ismayilova and Yaya (2023) Canada	To explore women's experiences managing PCOS and the barriers and facilitators they encountered in their management journeys	25 participants with a medical diagnosis of PCOS; Age ≥ 18 years	Black (4), East Asian (8), Middle Eastern (8), South Asian (8), and White/Caucasian (72)	Semi‐structured telephone interviews	Thematic analysis
Soucie et al. (2021) Canada	To examine the potential reasons underlying this diagnosis delay	62 participants had a PCOS diagnosis from a Canadian practitioner; Age over 18 years	White (82), Middle Eastern (6.5), South Asian (1.5), and mixed race (10)	Semi‐structured interview was held in a private research space on campus or on Skype	Reflexive thematic analysis
Crete and Adamshick (2011) The United States	To describe the lived experience of women with PCOS in the management of their disorder and the meaning of that experience for them	10 participants diagnosed with PCOS and managed within the past 5 years; Able to speak and understand English; 18 years or older	Caucasian (70), Native American (10) and Latina (20)	Semi‐structured interviews	Hermeneutic phenomenological reflection
Sydora et al. (2023) Canada	To assess the perceptions of health status, health care experience and disease management support in those affected by PCOS in Alberta, Canada	194 responses were included; Over 18 years; 187 participants diagnosed PCOS, 7 suspected to have PCOS	Caucasian/White (84.5), Asian (7.2), African/Black (3.1), Indigenous/First Nation (2.1) and other (3.1)	Online questionnaire, checklist, and open questions; Responses were collected online into the REDCap database	Descriptive statistics and thematic analyses

### 4.2 Thematic Analysis

#### 4.2.1 Thematic Heading 1: Responsive Care From Healthcare Practitioners

The healthcare experience of women with PCOS regarding responsive care from medical staff includes knowledge of healthcare practitioners (Crete and Adamshick 2011; Ismayilova and Yaya 2023, 2022a, 2022b; Tomlinson et al. 2017), information (Crete and Adamshick 2011; Ismayilova and Yaya 2023, 2022a, 2022b; Soucie et al. 2021; Sydora et al. 2023; Tomlinson et al. 2017), weight bias (Ismayilova and Yaya 2023, 2022a; Tomlinson et al. 2017), and apathetic healthcare professionals (Crete and Adamshick 2011; Ismayilova and Yaya 2023, 2022a, 2022b; Soucie et al. 2021; Sydora et al. 2023; Tomlinson et al. 2017). The knowledge of healthcare practitioners (HCP) in recognising and diagnosing PCOS is essential in helping women with PCOS confirm the diagnosis and make early interventions. However, the majority of primary care practitioners in clinical practice settings have limited knowledge of PCOS. Due to the diversity of symptoms of PCOS, it affects each individual differently and even manifests itself differently in different racial groups (Teede et al. 2023). This results in healthcare personnel being unable to recognise these symptoms and make further diagnoses in time for the patient's first visit, ultimately leading to a delay in diagnosis. One participant expressed how common physicians overlook PCOS conditions, leaving symptoms untreated and uncontrolled for years. Another participant expressed a desire for a PCOS diagnosis rather than vague answers from doctors. To receive a further diagnosis, these women who suffer from symptoms often have to visit several doctors before obtaining a diagnosis (Tomlinson et al. 2017). This inability to get a diagnosis timely in the first place makes numerous women with PCOS suspect the competence of their physicians, which affects their trust in healthcare practitioners (Ismayilova and Yaya 2022a).

A considerable gap in the provision of information by healthcare practitioners (Crete and Adamshick 2011; Ismayilova and Yaya 2023, 2022a, 2022b; Soucie et al. 2021; Sydora et al. 2023; Tomlinson et al. 2017). It is also the reason that causes PCOS women to become disappointed, frustrated, and disempowered with the management and treatment of PCOS, ultimately affecting PCOS women's trust in their physicians. One participant expressed ignorance of the potential complications and risk factors associated with PCOS (Ismayilova and Yaya 2022a, 12). Other participants mentioned never receiving any information related to PCOS from their healthcare professionals. They expressed a strong need and desire to obtain information about PCOS from healthcare providers. ‘I am craving information and feel like I have been hunting for answers for years. Still don't feel grounded in a treatment plan’ (Sydora et al. 2023, 9). ‘Because it's not that I wouldn't trust my doctor if they gave me that information but they don't’. (Ismayilova and Yaya 2023, 61).

Obesity is a common symptom of PCOS, and weight management is a headache for this population. However, weight bias was prevalent among healthcare workers for PCOS‐affected women. Healthcare providers always put their focus on the problem of overweight in women with PCOS when dealing with their other issues, as if everything will go back to normal only if their weight is under control. ‘You're overweight and that's the problem’ (Ismayilova and Yaya 2022a, 10). Doctors always informed PCOS women that they needed to lose weight with a dismissive attitude but never provided any suggestions on diet, nutrition, and lifestyle.

Apathetic and unsympathetic healthcare providers were identified in the studies. Women with PCOS often have a myriad of health concerns, but those are often disregarded and dismissed by healthcare workers, even as not worth mentioning. ‘One woman recalled that the first doctor she saw in university discounted her anxiety: “he treated it like it was nothing” (Soucie et al. 2021, 528)’. Doctors neglected the psychological and health concerns of women with PCOS, rarely communicated with them, and even excluded them from the consultation process (Sydora et al. 2023). ‘I had doctors who didn't believe me. I have a fertility specialist that laughed me out of their office. I feel it's pretty difficult when dealing with PCOS I have managed to completely ostracise myself in some ways because of having to go through some of the hurdles without any support’ (Sydora et al. 2023, 9). This inattentive, apathetic, and indifferent attitude often leaves PCOS women feeling abandoned, frustrated, and unwilling to seek help from healthcare practitioners anymore.

#### 4.2.2 Thematic Heading 2: Managing Polycystic Ovarian Syndrome

The experience of women with PCOS in managing polycystic ovarian syndrome involves the following subthemes: access to specialist care (Ismayilova and Yaya 2022b; Soucie et al. 2021; Sydora et al. 2023; Tomlinson et al. 2017), frustration (Crete and Adamshick 2011; Ismayilova and Yaya 2023), medication (Crete and Adamshick 2011; Soucie et al. 2021; Sydora et al. 2023; Tomlinson et al. 2017), fertility management (Crete and Adamshick 2011; Soucie et al. 2021; Sydora et al. 2023; Tomlinson et al. 2017), and information (Crete and Adamshick 2011; Ismayilova and Yaya 2022a; Sydora et al. 2023; Tomlinson et al. 2017).

Findings from four studies (Ismayilova and Yaya 2022b; Soucie et al. 2021; Sydora et al. 2023; Tomlinson et al. 2017) stressed that it is difficult for women with PCOS to obtain referrals for advice from specialists in the field. In many cases, they needed to urge their practitioners to make a referral for themselves (Soucie et al. 2021). However, doctors often reject such requests for various reasons, such as age factors, obscure symptoms, etc. (Tomlinson et al. 2017). Some participants complained that the doctor's attitude became cold and impatient whenever the request for referral was brought up, ‘he refuses to listen to my request for a referral’, and the topic was discussed at length and intensely (Sydora et al. 2023). Owing to the lack of timely access to specialist advice, plenty of PCOS women are not diagnosed until many years later (Sydora et al. 2023). ‘I started my periods at 15, and I thought I'd give it a little bit of time‐ but they never equaled out, and the weight gain started when I was about 18 or 19. I kept going back to my doctor, and they say you're too young to have that, don't be silly, yeah basically pushed out the door – ‘I'm a doctor I know your body better than you do’ and I've had to fight and fight and fight, and I finally got referred to an endocrinologist, who said ‘yep, they should have sent you to me years ago’ (Tomlinson et al. 2017, 2322).

The lack of information was identified in PCOS women in managing their symptoms (Crete and Adamshick 2011). Healthcare providers did not offer adequate information to women with PCOS to assist them in understanding the meaning and risk factors of PCOS, nor did they render any lifestyle changes or dietary modification programs to help them manage their symptoms. The lack of attention from healthcare workers was so frustrating for PCOS in managing their symptoms that they became confused and even lost hope in the management of PCOS. ‘The information that I've been looking for hasn't come from a GP. When I was diagnosed, I wasn't given anything. Like in that experience, that kind of leaves me with my GP knows nothing about it’ (Ismayilova and Yaya 2023, 62). They turned to the internet for self‐education. Nevertheless, the authenticity and credibility of the information on the internet are difficult to discern, and most of the time, they need to experiment with the information themselves before they can finally recognise its relevance, usefulness, or falsehood. ‘That's all they told me—that you have a potential for getting diabetes, they didn't really inform me about anything much. This is what I found out later on from the internet’ (Crete and Adamshick 2011, 261). ‘I trust the information in all of these books and looking at kind of what the popular opinion is on certain things. Like testing out all these different supplements and then trial and error myself’ (Ismayilova and Yaya 2023, 62). In contrast, when healthcare workers provided sufficient information and enough attention to PCOS women, it made them feel that they were supported and valued. ‘The information from my most recent GP that actually did the diagnosis was probably the most helpful to me based on the fact that it kind of spurred me to really look into and kind of understand something that I kind of didn't really take seriously before’ (Ismayilova and Yaya 2022a, 14).

Women with PCOS felt a great deal of despair in managing their symptoms (Ismayilova and Yaya 2023). This was because healthcare workers failed to explain the overall health meaning, complications, and potential risk factors of PCOS (e.g., diabetics, cardiovascular disease, mental health, etc.) as well as failed to render the approaches of symptom management. ‘They never told me. When I was diagnosed with PCOS, that yes, this is stopping you from conceiving. However, this is something that is part of your overall health just so you know this is something you have to keep watch of. It's going to cause different issues in your life. Never, never told me that’ (Crete and Adamshick 2011, 261).

Nearly every woman with PCOS who sought help from a healthcare worker for her irregular periods was prescribed birth control pills. It seems that the pill is the answer to all the problems PCOS creates. ‘One woman said her specialist “put me on birth control and that was his big answer, he said everything would go away with birth control” (Soucie et al. 2021, 528).’ Ironically, these women were not informed about the therapeutic principles and adverse effects of the pill, nor were they informed of the precautions to be taken. ‘She really didn't explain what is going to happen if I take this type medication, if I take that. I just wanted a little more information and I really didn't get it’ (Crete and Adamshick 2011, 262).

The management of infertility in women with PCOS is a significant challenge, as PCOS is considered one cause of female infertility (Santoro et al. 2016). This population presents significant challenges when dealing with the topic of infertility, as they have a more difficult time conceiving than ordinary healthy women and often require medical treatment to facilitate successful conception (Karjula et al. 2017). However, the risks of infertility associated with PCOS were rarely communicated to women with PCOS. ‘I had all the tests, they found I was ovulating and everything was sort of normal that way… First of all they said they thought one side was blocked and then they wrote to me again saying—“No, I think we got it wrong, you're fine” (Tomlinson et al. 2017, 2323)’. It's worth noting that when women with PCOS informed their physicians of their plans to conceive, they began to be taken seriously. ‘After I had mentioned to her that I wanted to have a baby, that's when I felt like she started to take me more seriously, not in regards to my symptoms, just in regards to I wasn't getting pregnant’ (Soucie et al. 2021, 527).

#### 4.2.3 Thematic Heading 3: Polycystic Ovary Syndrome and Its Impact on Self‐Image

The healthcare experience of women with PCOS surrounding PCOS and its impact on self‐image comprised symptoms (Crete and Adamshick 2011; Ismayilova and Yaya 2023, 2022a; Sydora et al. 2023; Tomlinson et al. 2017), fertility issues (Crete and Adamshick 2011; Ismayilova and Yaya 2023; Tomlinson et al. 2017), and emotional swings (Crete and Adamshick 2011; Ismayilova and Yaya 2023, 2022a; Soucie et al. 2021; Tomlinson et al. 2017).

Symptoms induced by PCOS put women with PCOS in a situation of self‐image disorders. These symptoms make them feel like they are monsters. ‘I still have a body image issue. You know how they call it the “bearded lady syndrome”’. ‘Sometimes I don't feel like explaining to everybody around me why I have this look. I don't feel like going through the ordeal of what the problem is’ (Crete and Adamshick 2011, 260). Furthermore, infertility issues cause women with PCOS to struggle with their sense of womanhood as they face challenges in conceiving (Ahmadi et al. 2020). Some participants reported experiencing mental health issues owing to infertility. ‘Being young and hearing having kids would be extremely difficult without help was extremely mentally challenging. I was put on anti‐depressants shortly after that’ (Sydora et al. 2023, 9). They expressed that it would be beneficial if healthcare workers could render explanations about infertility and inform them of the available support to address their concerns and doubts (Tomlinson et al. 2017).

Mental health issues such as mood swings are prevalent among women with PCOS (Crete and Adamshick 2011; Soucie et al. 2021). The causes of these mood swings were not limited to self‐image disturbances caused by the physical effects of PCOS, also reflected in interactions with healthcare workers and the misunderstanding of their friends and family members. ‘I wish she would have maybe talked about more like the risk. Like it's the higher risk for depression and anxiety, and that's something that I have trouble with. Particularly the anxiety. Or like I sort of mentioned before, the tendency to binge eat because I have that problem as well’ (Ismayilova and Yaya 2023, 12). Moreover, It was found that healthcare practitioners only began to take PCOS women seriously when they expressed a need to get pregnant (Sydora et al. 2023). However, the health needs of this population far outweighed the need to conceive, but healthcare professionals seemed to be invisible to them. ‘Treat women as more than child‐bearers. PCOS affects more than just ovaries. It also affects our mental health’ (Sydora et al. 2023, 11).

## 5 Discussion

### 5.1 Responsive Care From Healthcare Practitioners

The review showed that the topic of responsive care from healthcare practitioners included information availability, healthcare practitioners' attitudes, HCP knowledge, and weight bias (Crete and Adamshick 2011; Ismayilova and Yaya 2023, 2022a, 2022b; Soucie et al. 2021; Sydora et al. 2023; Tomlinson et al. 2017).

The study indicated that healthcare providers often fail to provide adequate information and resources to women with PCOS, and sometimes provide none at all (Crete and Adamshick 2011; Ismayilova and Yaya 2023, 2022a, 2022b; Soucie et al. 2021; Sydora et al. 2023; Tomlinson et al. 2017). This lack of support not only causes women with PCOS to lose confidence in treatment and feel helpless but also damages the relationship between women with PCOS and healthcare providers. Apart from that, healthcare practitioners often demonstrate insufficient ability to diagnose PCOS, a limited recognition of its symptoms, and inadequate knowledge about the condition (Ismayilova and Yaya 2022a, 2022b; Tomlinson et al. 2017). To meet the information needs of women with PCOS, Sydora et al. (2023) recommended distributing information about the causes, symptom management, complications, and treatments of PCOS to them in the form of brochures. To mitigate the knowledge gap among healthcare practitioners, studies advocate for training primary care providers, establishing PCOS‐specific clinics, fostering interdisciplinary teamwork, and promoting patient‐centered care (Crete and Adamshick 2011).

Another significant concern is the attitude of healthcare workers toward women with PCOS. Many participants reported that their doctors demonstrated a lack of empathy and patience, appeared indifferent to their health concerns or mental health needs, and often ignored their input—even when patients initiated discussions about their symptoms (Ismayilova and Yaya 2022a). In contrast, when healthcare providers showed enthusiasm, patience, and professionalism, patients reported feeling supported and cared for. Therefore, to improve healthcare practitioners' responsiveness, it is recommended to incorporate training on empathy and patient responsibility into medical education curricula (Sydora et al. 2023).

In addition, Ismayilova and Yaya (2022a) highlighted that weight bias is a common problem faced by women with PCOS when interacting with healthcare providers. PCOS symptoms often make it difficult for these women to avoid obesity, and weight loss or management may be more challenging for them compared to women without PCOS (Alur‐Gupta et al. 2019). However, doctors and others who were not unfamiliar with PCOS symptoms frequently attribute all health issues—such as infertility and menstrual irregularities—to obesity. They harshly and coldly told patients that these conditions were due to obesity and advised women with PCOS to lose weight but never provided specific weight loss advice or guidance, which leads to patients feeling frustrated and helpless (Ismayilova and Yaya 2023; Soucie et al. 2021; Tomlinson et al. 2017). In conclusion, a deeper understanding of the key issues that healthcare professionals face in responding to the needs of women with PCOS provides important insights for nursing practice. As a core force in the healthcare system, nurses can actively improve the management and care experience of women with PCOS in a variety of ways. First, women with PCOS can be helped to fill their knowledge gaps by distributing brochures on PCOS symptom management and treatment or by organizing regular group education activities. Secondly, when communicating with women with PCOS, we should maintain patience and respect, listen carefully to their complaints, pay attention to their mental health needs, and actively provide emotional support and necessary psychological intervention to enhance their trust and compliance. In addition, nurses should provide individualised nursing interventions to address weight.

### 5.2 Managing Polycystic Ovary Syndrome

The experiences of PCOS‐affected women were reflected not only in the healthcare staff's responses to care but also in the management of PCOS. These experiences encompassed five areas: access to specialist care, frustration, the use of medications, fertility, and information (Crete and Adamshick 2011; Ismayilova and Yaya 2023, 2022b; Soucie et al. 2021; Sydora et al. 2023; Tomlinson et al. 2017).

Studies have shown that PCOS patients have significant difficulties in obtaining professional healthcare (Ismayilova and Yaya 2022b; Sydora et al. 2023). This was mainly reflected in the fact that some doctors do not have enough knowledge of PCOS symptoms, and even when patients actively request a referral to endocrinology or gynaecology, doctors fail to respond promptly. Some doctors were hesitant or refused referral requests (Tomlinson et al. 2017). This delay in diagnosing PCOS may cause patients to miss the optimal window for early intervention. Therefore, it is particularly important to adopt an interdisciplinary team approach when managing the health status of patients with PCOS (Sydora et al. 2023).

In the management of PCOS, many women expressed deep frustration with healthcare practitioners and the care they provided. These frustrations were manifested in several aspects, such as symptom management, indifferent attitude from healthcare practitioners, choice of treatment, and the mismanagement of PCOS (Crete and Adamshick 2011). This negative sentiment further exacerbates tensions between doctors and patients. Moreover, due to limited medication options, adverse effects, and inadequate precautions, many women with PCOS find it difficult to effectively control their symptoms (Soucie et al. 2021). At the same time, the high cost of fertility treatment and repeated ovulation induction treatments also put heavy physical and mental pressure on patients (Tomlinson et al. 2017).

Due to the lack of comprehensive information about PCOS symptoms, further leaves women with PCOS feeling unsupported, often driving them to seek self‐education through the Internet (Sydora et al. 2023). However, the quality of information on the Internet varies greatly and lacks authority. Patients often need to verify the authenticity and usefulness of the information themselves (Ismayilova and Yaya 2023). In summary, the healthcare support that women with PCOS receive in symptom management is still very limited, and they urgently need more support and guidance from professional nurses. In the comprehensive management of PCOS, nurses should not only be information transmitters, but also play the role of emotional supporters and coordinators. Therefore, nurses should identify the needs of women with PCOS who have complex or persistent symptoms during their initial contact and actively assist them in obtaining timely referral and interprofessional support. Secondly, in response to the disappointment and helplessness of women with PCOS during the treatment process, nurses should show more empathy, improve the quality of communication, and relieve tension. Finally, nurses can address information gaps by providing educational manuals or organizing health education courses to help women acquire accurate management knowledge. Through these measures, nurses can significantly improve the symptom control ability and overall quality of life of women with PCOS.

### 5.3 Polycystic Ovary Syndrome and Its Impact on Self‐Image

PCOS had a profound and complex impact on women's mental and physical health, seriously undermining patients' self‐image and self‐esteem. Among them, infertility was considered one of the most challenging problems faced by women with PCOS (Crete and Adamshick 2011; Sydora et al. 2023; Tomlinson et al. 2017). For women who had PCOS, infertility was not only a physical obstacle, but also a heavy psychological burden, which profoundly affected their sense of shame, identity, and the cognition and shaping of femininity (Crete and Adamshick 2011). This psychological impact was often intertwined with cultural background and social expectations, leaving patients feeling isolated and helpless. Furthermore, women who had PCOS often faced tremendous financial pressure and psychological burden during repeated attempts at fertility treatment. This double pressure not only undermined their confidence and expectations of a successful pregnancy but also led to significant physical and psychological stress. This vicious cycle left many women feeling helpless and hopeless, affecting their overall quality of life. Worryingly, when women with PCOS sought healthcare, their self‐image needs and mental health were often overlooked. Some patients reported being treated coldly or laughed at, and this inappropriate behaviour further exacerbated their emotional fluctuations and psychological burden (Sydora et al. 2023). This inappropriate healthcare experience not only undermined patients' trust in the healthcare system but also hindered them from receiving timely and effective treatment. Thus, it was crucial to improve the healthcare experience and psychological support for women who had PCOS. It required healthcare education, interdisciplinary team collaboration, and mental health intervention to more comprehensively meet the needs of women with PCOS and help them rebuild their self‐confidence and positive self‐cognition, thereby improving their overall health and quality of life.

This systematic review has several limitations. First, since all studies were from developed countries, the findings may not be generalisable. Secondly, the participants in the included studies were selected through convenience and purposive sampling methods, which in itself limited the representativeness of the sample, meaning that not every woman with PCOS had an equal opportunity to participate in the study. Therefore, this may have led to a certain bias among the researchers. Finally, this study only retrieved English‐language articles, which may have led to searching and reporting bias. It is recommended that future reviews include more articles published in other languages to more fully reflect the global nature and diversity of nursing knowledge.

## 6 Conclusion

The results of this review exhibited that women with PCOS often encounter negative healthcare experiences (e.g., difficulties with referrals, scepticism about doctors' knowledge of PCOS, lack of authoritative information, concerns about the impact of medication, infertility issues, and the indifference of healthcare workers), which undermine their trust in healthcare workers, reduce their sense of well‐being, and may lead to tension in the doctor–patient relationship. Evidence showed that the development of a multidisciplinary PCOS clinic, the establishment of online support groups, and the creation of comprehensive patient‐centered treatment plans can help women with PCOS resolve healthcare problems. Furthermore, the care and support of healthcare workers are crucial. Doctors should pay more attention to the mental health of PCOS women, assess anxiety and depression, and intervene as early as possible to prevent mental problems from worsening.

## Author Contributions

Baoying Zhang and Joan Lalor designed the study. Baoying Zhang performed the literature search, screened the studies, and extracted the data. Baoying Zhang and Joan Lalor wrote and reviewed the manuscript.

## Conflicts of Interest

The authors declare no conflicts of interest.

## Data Availability

The authors have nothing to report.
